# Evaluation of the Mechanical Behavior of the Patellar and Semitendinosus Tendons Using Supersonic Shear-wave Imaging (SSI) Elastography and Tensile Tests

**DOI:** 10.1055/s-0044-1788290

**Published:** 2024-09-04

**Authors:** André Fontenelle, Pietro Mannarino, Liliam Fernandes de Oliveira, Luciano Luporini Menegaldo, Sérgio Augusto Lopes de Souza, César Rubens da Costa Fontenelle

**Affiliations:** 1 Serviço de Traumato-Ortopedia, Hospital Universitário Clementino Fraga Filho, Universidade Federal do Rio de Janeiro, Rio de Janeiro, RJ, Brasil; 2 Departamento de Ortopedia e Traumatologia, Faculdade de Medicina, Universidade Federal do Rio de Janeiro, Rio de Janeiro, RJ, Brasil; 3 Programa de Engenharia Biomédica, Instituto Alberto Luiz Coimbra de Pós-Graduação e Pesquisa de Engenharia (Coppe), Universidade Federal do Rio de Janeiro, Rio de Janeiro, RJ, Brasil; 4 Departamento de Radiologia, Faculdade de Medicina, Universidade Federal do Rio de Janeiro, Rio de Janeiro, RJ, Brasil

**Keywords:** elasticity imaging techniques, patellar tendon, Young's modulus

## Abstract

**Objective** To analyze the mechanical properties of the patellar (PT) and semitendinosus (ST) tendons from fresh-frozen human cadavers from a tissue bank using supersonic shear-wave imaging (SSI) elastography and tensile tests.

**Methods** We tested seven PT and five ST samples on a traction machine and performed their simultaneous assessment through SSI. The measurements enabled the comparison of the mechanical behavior of the tendons using the stress x strain curve and shear modulus (μ) at rest. In addition, we analyzed the stress x μ relationship under tension and tested the relationship between these parameters. The statistical analysis of the results used unpaired *t* -tests with Welch correction, the Pearson correlation, and linear regression for the Young modulus (E) estimation.

**Results** The μ values for the PT and ST at rest were of 58.86 ± 5.226 kPa and 124.3 ± 7.231 kPa respectively, and this difference was statistically significant. The correlation coefficient between stress and μ for the PT and ST was very strong. The calculated E for the PT and ST was of 19.97 kPa and 124.8 kPa respectively, with a statistically significant difference.

**Conclusion** The ST was stiffer than the PT in the traction tests and SSI evaluations. The μ value was directly related to the stress imposed on the tendon.

**Clinical relevance** The present is an evaluation of the mechanical properties of the tendons most used as grafts in knee ligament reconstruction surgeries.

## Introduction

In recent decades, the mechanical properties of tendons have been widely studied, providing knowledge about their behavior. 1 2 3 4 Recently, the evaluation of the elastic modulus (E) and resistance to failure (RF) of tendon tissues gained importance in the literature. 5 In 2013, LaCroix et al. 5 showed an intimate relationship between these properties despite their conceptual distinction.

Publications on the matter employed several methods to measure these properties, but most carry biases. 3 6 7 8 9 10 11 12 13 14 The results of in-vivo studies are usually obtained indirectly from calculations combining magnetic resonance imaging and ultrasound with data provided by a dynamometer. 10 13 Several authors 2 3 6 9 11 12 14 have performed ex-vivo analyses of tendons from human and animal cadavers. However, the reliability of these studies is questionable due to the protocol preparation of cadaveric tissues. 10 15

We need new analysis methods including in-vivo studies and direct evaluation. Recently, ultrasound with elastography has been widely applied to evaluate tissue stiffness and measure it non-invasively, in real-time, and with less dependency on the operator's skill. 7 16 17 18 19 20

There are different elastography modalities, and the most recent is supersonic shear-wave imaging (SSI). 17 20 21 In this method, an acoustic radiation force generates shear waves in the tissue for detection by the ultrasound transducer. 14 17 21 The system provides the speed of these waves and the shear modulus (μ) of the tissue, expressing its stiffness. 12 14 17 19 21

The SSI is well-established in the evaluation of isotropic media, such as breast, liver, and thyroid tissues. 22 23 24 Despite requiring more robust validation, SSI has been used in musculoskeletal tissue, an anisotropic tissue, since the beginning of the last decade, with encouraging results. 1 6 7 8 16 18 19 20 25 26 27 Due to its anisotropic characteristic, the μ value of the tendon does not present the expected mathematical relationship with E. 14 17 21 Even so, recent studies 8 9 11 12 14 25 have demonstrated a strong correlation between the μ of the tendon obtained by SSI and its E calculated by the linear phase of the stress x strain graph. However, most of these tests used tendons from other animal species, with low scientific evidence. 9 11 12 14

There are two studies 8 25 in the literature comparing ex-vivo mechanical behaviors of human tendons using SSI elastographic analysis. The present study aims to evaluate the mechanical properties of the patellar (PT) and semitendinosus (ST) tendons due to their great relevance and use as grafts in knee ligament reconstruction surgeries 28 using SSI and dynamometry.

The main objective was to obtain and compare the E value from PT and ST at stress by evaluating the stress x strain curve recorded by the traction machine. The secondary objectives were to obtain and compare the μ values of PT and ST in the ultrasound assessment at rest carried out with SSI and to evaluate the influence of tissue stress in SSI-based evaluation, analyzing the correlation between μ values and the stress imposed on these tendons.

## Materials and Methods

### Ethical approval and study design

The institutional Ethics in Research Committee approved the present cross-sectional study under consubstantiated opinion 1,674,064 (CAAE: 26828914.3.0000.5257). The study was conducted at the Biomechanics Laboratory from 2019 to 2022.

The initial sample was composed of 14 PT and 19 ST samples from fresh-frozen human cadavers provided by the Brazilian Ministry of Health (MH) tissue bank. We received these tendons after they were considered unsuitable for surgical use, mainly due to contamination at some processing stage in the tissue bank. For the most part, the organism detected was *Staphylococcus epidermidis* , a bacterium that commonly colonizes human skin.

Tables 1 and 2 show the demographic distribution of the samples.

**Table 1:** Patellar tendon: demographic distribution

Gender	N	Mean age (years)
Male	5	28.2 (22–35)
Female	2	28 (26–30)

**Table 2:** Semitendinosus tendon: demographic distribution

Gender	N	Mean age (years)
Male	4	27.5 (22–35)
Female	1	22

The fact that tissues preserved in formaldehyde lose their mechanical properties warrants using fresh-frozen human tendons. 2 15 As such, we asked for samples from the MH since its protocol for preparing musculoskeletal tissues does not involve irradiation, avoiding damage to their biomechanical characteristics.

The inclusion criteria were tendons from fresh-frozen human cadavers aged between 20 and 35 years at the date of death and preserved in a freezer at -80° C at the tissue bank.

The exclusion criteria were signs of degenerative tendon disease, storage time longer than 2 years, presence of macroscopic ruptures, tendon irradiation during preparation by the tissue bank, and inadequate biomechanical or elastographic recording.

After applying the exclusion criteria and the occurrence of accidental damage to the tendons during the pretest preparation, we lost 7 PT and 14 ST samples, and 7 PT and 5 ST samples remained for the final analysis.

### Sample preparation

We kept the samples in a freezer at -20° C at the Immunology Laboratory. For testing, the tendons were thawed one hour before at room temperature.

**Fig. 1:**
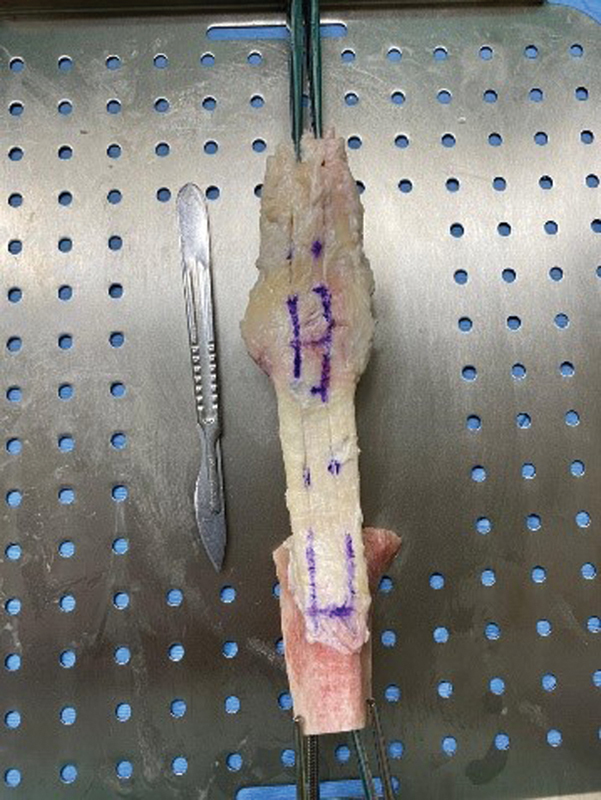
Patellar tendon (PT) prepared for the test.

Preparation of the PT started after thawing the anatomical piece provided by the MH, which consisted of the entire knee extensor apparatus, with the quadriceps tendon, patella, patellar tendon, and tibial tuberosity. The pieces for testing were prepared with bone plugs in both PT attachments, with approximately 1.0 cm in each dimension. The intermediate tendinous part was approximately 1.0 cm wide ( Fig. 1 ). Each bone plug was drilled with a 2.5-mm drill, generating holes for passing Ethibond 5 wire (Ethicon, Inc., Raritan, NJ, United States).

Processing of the ST occurred before freezing in the tissue bank. Its length was standardized from the myotendinous junction to its tibial attachment. After thawing, we performed a Krakow suture with Ethibond 5 at both ends to optimize anchorage.

**Fig. 2:**
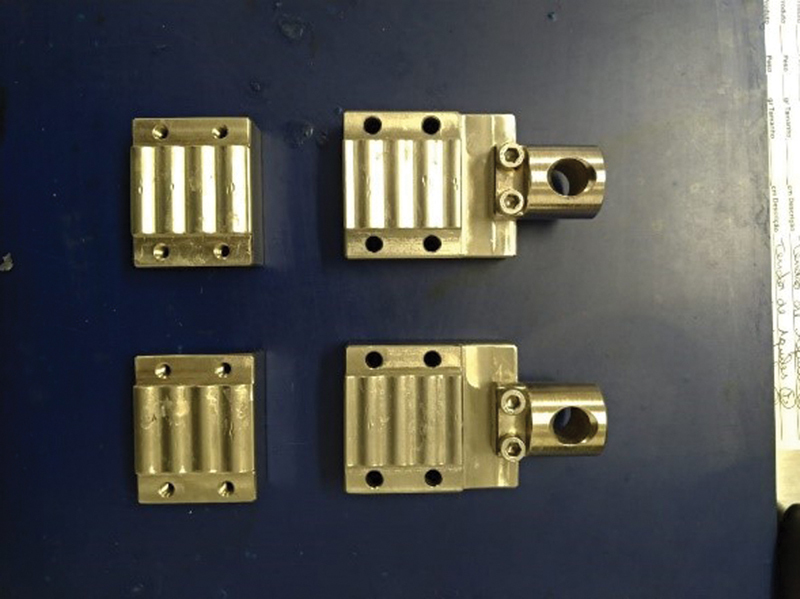
Metal fixing claws.

Both tendons were placed in the fixation system of the universal testing machine using metal claws ( Fig. 2 ).

**Fig. 3:**
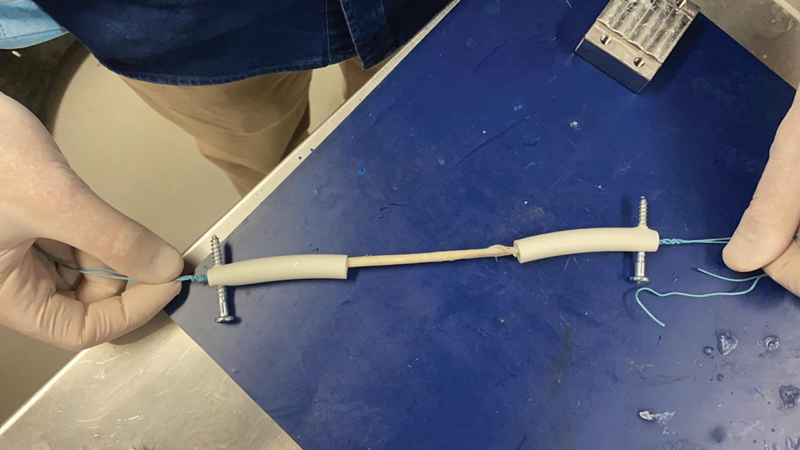
Semitendinosus tendon (ST) inserted into tubes and fixated to the framework with screws.

For the ST, we made two attempts before the definitive fixation to the metal claws. In the first attempt, the tendons were attached directly to the claws, which caused macroscopic structural damage in the first samples and loosening of their ends at the interface with the claws when the tensile test was still beginning. As a result, we abandoned this method. In the second attempt, we anchored the tendons by tying Ethibond 5 directly to plastic tubes attached to the claws. Once again, there were losses due to structural damage to the tissues and the generation of inadequate elastographic or biomechanical readings, so thbis method was also abandoned. After test failures with these two anchoring prototypes, we unfortunately lost 14 ST samples. Finally, to generate more effective fixation between the claw and the ST, we inserted them into plastic tubes, fixated them with Ethibond 5 threads, and attached them with conventional screws to a framework ( Figs. 3 4 ). This last method, deemed ideal and definitive, did not cause any damage or interference in data acquisition.

**Fig. 4:**
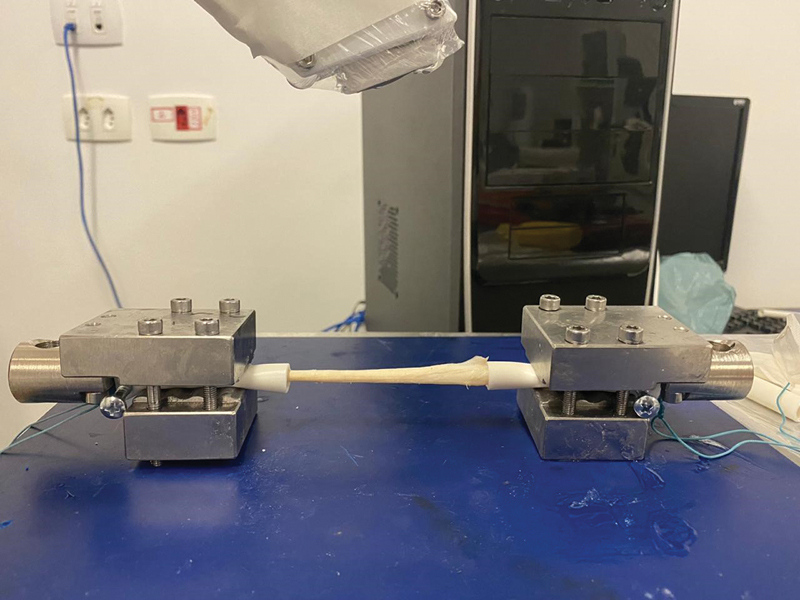
Final assembly of the ST.

### Elastography

**Fig. 5:**
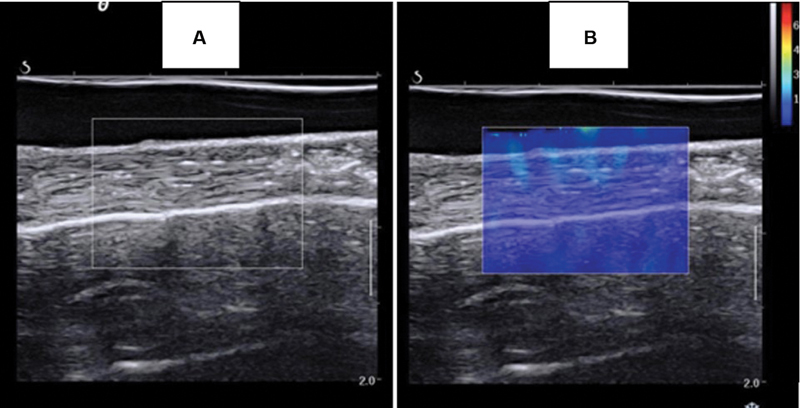
*.* ( **A** ) Tendon aligned in ultrasound (US) mode B. ( **B** ) Region of interest (ROI) in the elastography mode.

We used the Aixplorer equipment (SuperSonic Imagine, Aix-en-Provence, France) to acquire elastographic images with a linear transducer operating at frequencies ranging from 6 to 20 MHz. Before each test, we carefully aligned the transducer in the same direction as the tendon fibers using B-mode ultrasound ( Fig. 5A ), ensuring μ value determination in the same direction as that of the longitudinal traction.

We activated the elastographic mode using the adapted musculoskeletal (MSK) preset, whose scale ranges from 0 to 800 kPa. The mapping area had a rectangular shape, enabling tendon delimitation. The test started after 10 seconds to stabilize the color mapping of the elastographic images ( Fig. 5B ).

**Fig. 6.**
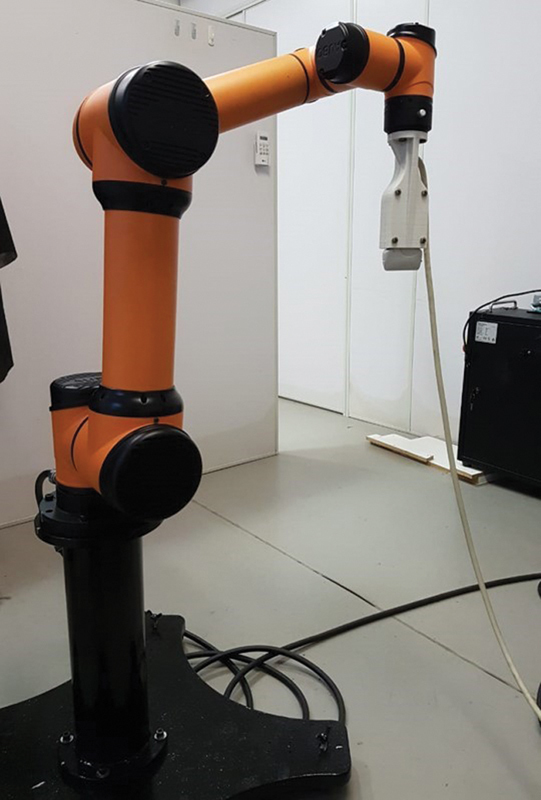
Robotic arm maintaining linear transducer positioning.

We used an AUBO i5 robotic arm (AUDO Robotics, Beijing, China) ( Figs. 6 7 ) to collect the images, keeping the transducer fixed and immobile over the region of interest (ROI). A gel (Ultrex-gel, Farmativa Indústria e Comércio Ltda., Rio de Janeiro, RJ, Brazil) was used for the acoustic coupling on the tendon surface.

**Fig. 7:**
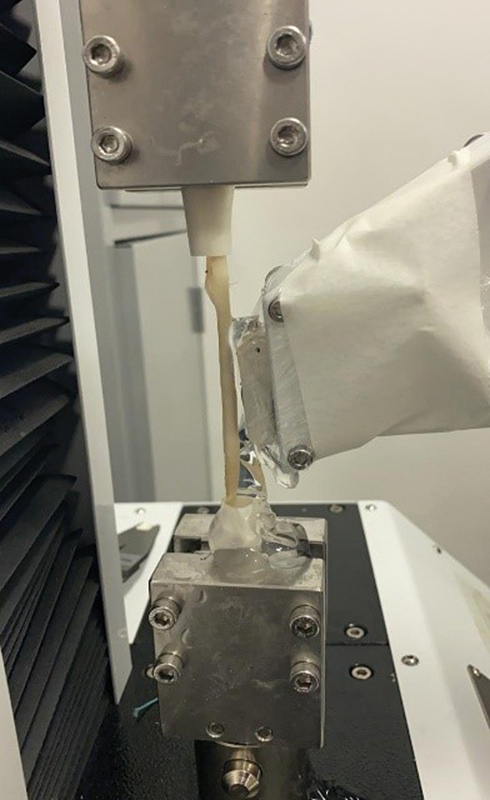
Transducer positioning during testing on the traction machine.

The μ analysis employed a specific routine developed by the Biomechanics Laboratory (trough the Matlab R2013a software, The MathWorks, Inc., Natick, MA, United States). We acquired elastography images until mapping saturation. At that moment, we terminated the video and stress.

### Biomechanical testing

We used a BioPDI (São Carlos, SP, Brazil) tensile testing machine with a 5-kN load cell to perform tensioning tests. The temperature and relative humidity in the laboratory during the tests were standardized and kept constant at 23° C and 50% respectively. We attached the ends of the PT and ST samples to the metal claws from the tensioning machine. One end remained fixed, while the other end was progressively pulled at a 1-mm/minute speed on a uniaxial longitudinal axis.

The testing machine values consist of position (mm) and force (N). Using the Matlab software, data underwent a sixth-order Butterworth filtering. We performed an exponential third-order adjustment, ending with 0.5 splines. After the process, we calculated the stress and strain using the previously-measured initial length and cross-sectional area.

### Statistical analysis

We calculated descriptive data, such as mean and standard deviation (SD). The Shapiro-Wilk test determined the distribution normality. The *t* -test for independent groups with Welch correction compared μ values at rest in the PT and ST samples. The Pearson correlation coefficient defined the relationship between μ under stress and tendon stress at moments of the stress x strain curve. Linear regression was performed on the distributions to measure the slope of the curve. E calculation used the slope ^-1^ formula, since stress was selected as the independent variable on the x-axis. Values of *p* < 0.05 were considered significant. The analyses were performed using the GraphPad Prism (GraphPad Software, Inc., La Jolla, CA, United States) software, version 7.0.

## Results

### Shear modulus at rest

**Fig. 8:**
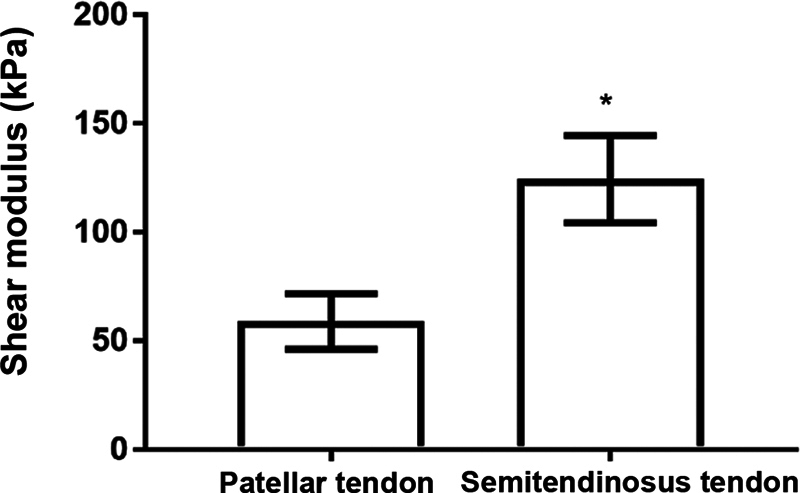
Shear modulus (μ) of the PT and ST at rest. **Note:** * *p* < 0.05.

The initial μ value (with no stress) of the ST was higher compared with that of the PT, which was statistically significant (ST = 124.3 ± 7.231 kPa; and PT = 58.86 ± 5.226; *p* = 0.0059) ( Fig. 8 ).

### Correlation between shear modulus and stress

The μ value presented a very strong correlation with stress for both tendons (PT: R = 0.9507; *p* < 0.0001; and ST: R = 0.9528; *p* < 0.0001) ( Table 3 ).

**Table 3:** Shear modulus (μ) x stress correlation

Correlation coefficient (R)	N	μ x *stress*	*p*
Patellar tendon	7	0.9507 (0.839–0.985)	< 0.0001
Semitendinosus tendon	5	0.9528 (0.845–0.986)	< 0.0001

### Variation of shear modulus under stress

**Fig. 9:**
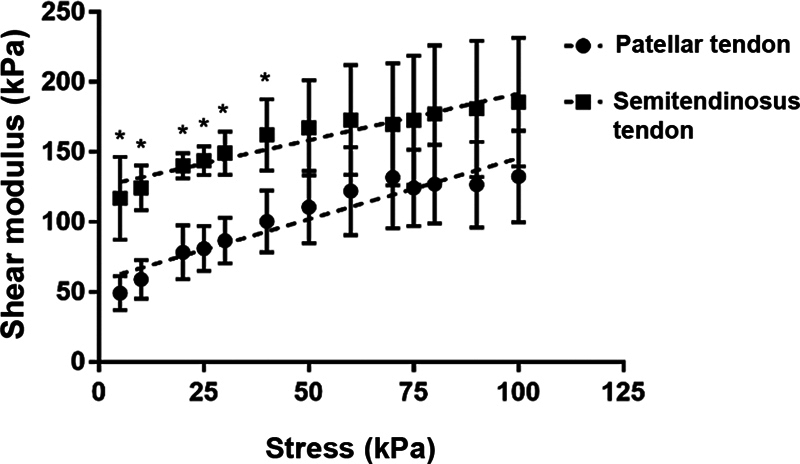
Stress x shear modulus (µ) relationship. **Note:** * *p* < 0.05.

The variation in μ values from ST and TP under stress presented no statistically significant difference (ST: slope of 0.664 ± 0.063 kPa; and PT: 0.872 ± 0.085 kPa; *p* = 0.065). However, there was a statistically significant difference regarding the μ values of the tendons, especially noted at lower stresses (ST: an increase of 116.8–133.3 kPa; and PT: an increase of 47.14–69.31 kPa; *p* < 0.0001) ( Fig. 9 ).

### Percentage of strain under stress and Young modulus

**Fig. 10:**
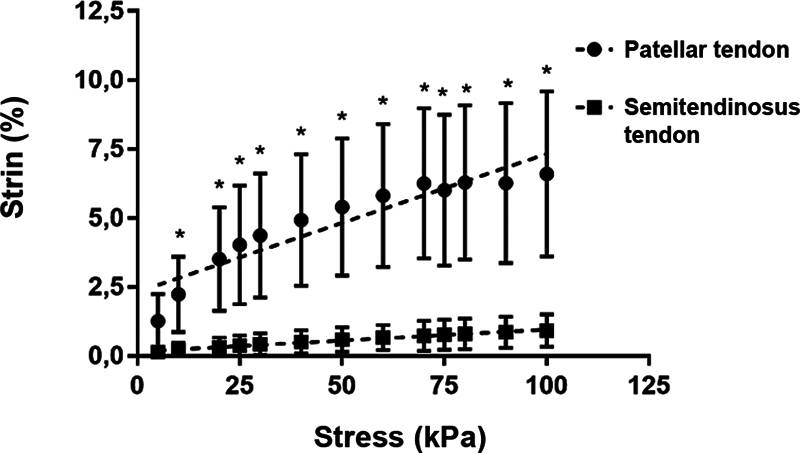
Stress x strain relationship. **Note:** * *p* < 0.05.

The ST showed significantly higher resistance to deformation than PT (ST: slope of 0.05 ± 0.005; and PT: slope of 0.008 ± 0.0002; *p* < 0.0001). There was a statistically significant difference between the calculated E of the tendons (ST = 124.8 kPa; and PT = 19.97 kPa; *p* < 0.0001) ( Fig. 10 ).

## Discussion

Studying the mechanical properties of tendons provides information about their function and disease prevention and treatment. 1 3 18 26 27 29 In this context, the ST and PT are critical due to their wide use as grafts in surgeries for ligament reconstruction. 28

The present study used tendons from fresh-frozen cadavers provided by the MH, not damaged by formaldehyde or ionizing radiation, enabling the evaluation of the stress x strain curve and computation of the relationship between the stress and the strain. The ST showed significantly higher resistance to strain under stress than the PT ( *p* < 0.0001). We also observed a significant difference for the calculated E, showing a much higher value in the ST (ST = 124.8 Kpa; and PT = 19.97 Kpa; *0* < 0.0001). We compared μ values from the PT and ST obtained through ultrasound with SSI assessment at rest. Again, the ST was stiffer than the PT (ST = 124.3 ± 7.231 kPa; and PT = 58.86 ± 5.226 Kpa; *p* = 0.0059), showing agreement between the results obtained with SSI and those from the tensile tests. We also recorded the influence of tissue stress on the SSI assessment, showing that the greater the stress applied, the greater the μ recorded in both tendons, with a very strong correlation between the parameters (PT: R = 0.9507; *p* < 0.0001; and ST: R = 0.9528; *p* < 0.0001).

In the last decades the relevance of the SSI in real-time in-vivo tendon evaluation has been demonstrated. 7 18 19 20 26 27 29 A series of studies 19 29 30 have drawn attention to the importance of standardizing image acquisition, especially regarding the position of the limb examined. In 2019, for the first time, a study 8 showed the intimate relationship between the μ recorded using SSI and the stress imposed on a non-formolized human tendon; however, this work used a single specimen, presenting a low level of evidence. In the present study, we repeated this testing with two types of tendons (PT and ST), using 12 samples, showing a very strong and statistically significant correlation between these two variables. This sheds new light on the use of tendon assessment with SSI. From this moment on, it becomes critical to control the muscular action over the tendon studied, as this can significantly affect the elastographic record.

The current literature 9 12 consistently demonstrates the relationship between E and μ, although not respecting the classic mathematical estimate. Recently, in 2023, Brandão et al. 25 studied 5 PTs and 11 calcaneus tendons from fresh-frozen human cadavers, finding a strong correlation between the variation in μ recorded by SSI with the E calculated by the stress x strain curve in the biomechanical test. In the present study, stiffness was higher in the ST than in the PT, both in the SSI and the traction test, with statistical significance. This suggests that SSI can reliably measure the mechanical properties of tendons in a way comparable to the gold standard.

In the context of using the PT and ST as grafts for ligament reconstruction, it is interesting that these tendons not only present high RF but also elastic characteristics similar to those of the native ligament. Previous studies 5 11 have revealed a statistically significant correlation between μ and RF in normal animal tendons. Likewise, a reduction in μ and RF has been described in diseased or chemically-damaged tendons. 11 14 Therefore, SSI may be particularly useful in assessing tendons and decision-making for graft selection. However, none of these studies performed such tests on human tendons.

The findings of the present study are consistent with those of Fontenelle et al. 19 who, in 2018, reported that the in-vivo μ value of the ST was higher than that of the PT in relaxed and stressed states. This may suggest the choice of the ST when reconstructing a more rigid structure.

It is worth highlighting certain limitations of the present study. Although we obtained consistent E values by calculating the slope of the stress x strain curve, 19 the trendons were not brought to failure. The tests were interrupted when the SSI reached saturation, and the region of elastic strain of the tendon may not have been reached, which would compromise the E estimate. Furthermore, despite all care, tendon fixation to the metal claws of the traction machine was particularly difficult when there was no bone plug. This difficulty can generate micromovements at the tendon-claw interface, underestimating the deformation record obtained. Future studies must consider this.

Finally, the most appropriate graft depends on the mechanical behavior not only of the tendon, but also of the ligaments for replacement. Future research in this field should pay particular attention to the biomechanical analysis of these ligaments.

## Conclusion

The ST was stiffer than the PT both in the traction test and SSI evaluation at rest and under stress, with μ values revealing a direct relationship with the stress imposed on the tendon during its assessment.

## References

[1] Uehara H Itoigawa Y Wada T Shear wave elastography correlates to degeneration and stiffness of the long head of the biceps tendon in patients undergoing tenodesis with arthroscopic shoulder surgery J Shoulder Elbow Surg 2024 33 01 e31 e41 37327988 10.1016/j.jse.2023.05.014

[2] Woo S L Orlando C A Camp J F Akeson W H Effects of postmortem storage by freezing on ligament tensile behavior J Biomech 1986 19 05 399 404 3733765 10.1016/0021-9290(86)90016-3

[3] Mert A Cinaroglu S Keleş H Aydin M Çiçek F Evaluation of Autografts Used in Anterior Cruciate Ligament Reconstruction in Terms of Tensile Strength Cureus 2023 15 06 e39927 37409216 10.7759/cureus.39927 PMC10318378

[4] Nagelli C V Hooke A Quirk N Mechanical and strain behaviour of human Achilles tendon during in vitro testing to failure Eur Cell Mater 2022 43 153 161 35446434 10.22203/eCM.v043a12 PMC9286485

[5] LaCroix A S Duenwald-Kuehl S E Lakes R S Vanderby R Jr Relationship between tendon stiffness and failure: a metaanalysis J Appl Physiol 2013 115 01 43 51 23599401 10.1152/japplphysiol.01449.2012 PMC3727010

[6] Götschi T Schärer Y Gennisson J L Snedeker J G Investigation of the relationship between tensile viscoelasticity and unloaded ultrasound shear wave measurements in ex vivo tendon J Biomech 2023 146 111411 36509025 10.1016/j.jbiomech.2022.111411

[7] Mannarino P Lima K MM Fontenelle C RC Analysis of the correlation between knee extension torque and patellar tendon elastic property Clin Physiol Funct Imaging 2018 38 03 378 383 28707752 10.1111/cpf.12424

[8] Ahmadzadeh S MH Chen X Hagemann H Tang M X Bull A MJ Developing and using fast shear wave elastography to quantify physiologically-relevant tendon forces Med Eng Phys 2019 69 116 122 31056401 10.1016/j.medengphy.2019.04.005

[9] Zhang Z J Fu S N Shear Elastic Modulus on Patellar Tendon Captured from Supersonic Shear Imaging: Correlation with Tangent Traction Modulus Computed from Material Testing System and Test-Retest Reliability PLoS One 2013 8 06 e68216 23826378 10.1371/journal.pone.0068216 PMC3695046

[10] Bachmann E Rosskopf A B Götschi T T1- and T2*-Mapping for Assessment of Tendon Tissue Biophysical Properties: A Phantom MRI Study Invest Radiol 2019 54 04 212 220 30444794 10.1097/RLI.0000000000000532

[11] Martin J A Biedrzycki A H Lee K S In Vivo Measures of Shear Wave Speed as a Predictor of Tendon Elasticity and Strength Ultrasound Med Biol 2015 41 10 2722 2730 26215492 10.1016/j.ultrasmedbio.2015.06.008 PMC4556570

[12] Rosskopf A B Bachmann E Snedeker J G Pfirrmann C WA Buck F M Comparison of shear wave velocity measurements assessed with two different ultrasound systems in an ex-vivo tendon strain phantom Skeletal Radiol 2016 45 11 1541 1551 27631078 10.1007/s00256-016-2470-z

[13] Seynnes O R Kamandulis S Kairaitis R Effect of androgenic-anabolic steroids and heavy strength training on patellar tendon morphological and mechanical properties J Appl Physiol 2013 115 01 84 89 23620489 10.1152/japplphysiol.01417.2012

[14] Yeh C L Kuo P L Gennisson J L Brum J Tanter M Li P C Shear Wave Measurements for Evaluation of Tendon Diseases IEEE Trans Ultrason Ferroelectr Freq Control 2016 63 11 1906 1921 27824567 10.1109/TUFFC.2016.2591963

[15] Hohmann E Keough N Glatt V Tetsworth K Putz R Imhoff A The mechanical properties of fresh versus fresh/frozen and preserved (Thiel and Formalin) long head of biceps tendons: A cadaveric investigation Ann Anat 2019 221 186 191 29879483 10.1016/j.aanat.2018.05.002

[16] Dickson D M Fawole H O Newcombe L Smith S L Hendry G J Reliability of ultrasound strain elastography in the assessment of the quadriceps and patellar tendon in healthy adults Ultrasound 2019 27 04 252 261 31762782 10.1177/1742271X19859380 PMC6851722

[17] Taljanovic M S Gimber L H Becker G W Shear-Wave Elastography: Basic Physics and Musculoskeletal Applications Radiographics 2017 37 03 855 870 28493799 10.1148/rg.2017160116 PMC5452887

[18] Fontenelle C RDC Schiefer M Mannarino P Elastographic analysis of the supraspinatus tendon in different age groups Acta Ortop Bras 2020 28 04 190 194 32788862 10.1590/1413-785220202804229355 PMC7405844

[19] Fontenelle C RC Mannarino P Ribeiro F BDO Semitendinosus and patellar tendons shear modulus evaluation by supersonic shearwave imaging elastography Clin Physiol Funct Imaging 2018 38 06 959 964 10.1111/cpf.12506 29411519

[20] Lin D J Burke C J Abiri B Babb J S Adler R S Supraspinatus muscle shear wave elastography (SWE): detection of biomechanical differences with varying tendon quality prior to gray-scale morphologic changes Skeletal Radiol 2020 49 05 731 738 31811348 10.1007/s00256-019-03334-6

[21] Lima K MME Costa Júnior J FS Pereira W CA Oliveira L F Assessment of the mechanical properties of the muscle-tendon unit by supersonic shear wave imaging elastography: a review Ultrasonography 2018 37 01 3 15 28607322 10.14366/usg.17017 PMC5769952

[22] Barr R G Nakashima K Amy D WFUMB guidelines and recommendations for clinical use of ultrasound elastography: Part 2: breast Ultrasound Med Biol 2015 41 05 1148 1160 25795620 10.1016/j.ultrasmedbio.2015.03.008

[23] Ferraioli G Filice C Castera L WFUMB guidelines and recommendations for clinical use of ultrasound elastography: Part 3: liver Ultrasound Med Biol 2015 41 05 1161 1179 25800942 10.1016/j.ultrasmedbio.2015.03.007

[24] Cosgrove D Barr R Bojunga J WFUMB Guidelines and Recommendations on the Clinical Use of Ultrasound Elastography: Part 4. Thyroid Ultrasound Med Biol 2017 43 01 4 26 27570210 10.1016/j.ultrasmedbio.2016.06.022

[25] Clara A Brandão M Teixeira G C Rubens C Fontenelle C Fontenelle A Oliveira L F Menegaldo L L Correlation between the shear modulus measured by elastography (SSI) and tangent modulus from tensile tests of in vitro fresh-frozen human tendons J Biomech 2023 160 111826[published online ahead of print, 2023 Oct 5] 37826956 10.1016/j.jbiomech.2023.111826

[26] Mifsud T Chatzistergos P Maganaris C Supersonic shear wave elastography of human tendons is associated with in vivo tendon stiffness over small strains J Biomech 2023 152 111558 37004390 10.1016/j.jbiomech.2023.111558

[27] Akazawa T Miyamoto N Nishio H Age-related changes in mechanical properties of semitendinosus tendon used for anterior cruciate ligament reconstruction J Orthop Surg Res 2022 17 01 501 36403051 10.1186/s13018-022-03395-9 PMC9675133

[28] Widner M Dunleavy M Lynch S Outcomes Following ACL Reconstruction Based on Graft Type: Are all Grafts Equivalent? Curr Rev Musculoskelet Med 2019 12 04 460 465 31734844 10.1007/s12178-019-09588-w PMC6942094

[29] Mannarino P Matta T TD Oliveira L F An 8-week resistance training protocol is effective in adapting quadriceps but not patellar tendon shear modulus measured by Shear Wave Elastography PLoS One 2019 14 04 e0205782 30990803 10.1371/journal.pone.0205782 PMC6467440

[30] Berko N S Mehta A K Levin T L Schulz J F Effect of knee position on the ultrasound elastography appearance of the patellar tendon Clin Radiol 2015 70 10 1083 1086 26264499 10.1016/j.crad.2015.06.100

