# Supplementary material for: Evaluation of the Mechanical Behavior of the Patellar and Semitendinosus Tendons Using Supersonic Shear-wave Imaging (SSI) Elastography and Tensile Tests
Source: Rev Bras Ortop (Sao Paulo). 2024 Sep 4;59(4):e556–63. doi: 10.1055/s-0044-1788290 (PMC11374394; doi:10.1055/s-0044-1788290)
Supplement: Related texts

# Research article: Avaliação do comportamento mecânico dos tendões patelar e semitendinoso utilizando a elastografia por ondas de cisalhamento (SSI) e testes de tração

**Keywords:** módulo de Young, técnicas de imagem por elasticidade, tendão patelar

## Abstract

**Objetivo** Analisar as propriedades mecânicas dos tendões patelar (TP) e semitendinoso (ST) obtidos de cadáveres humanos congelados enquanto ainda frescos, provenientes de banco de tecidos, utilizando elastografia por ondas de cisalhamento ( *supersonic shearwave imaging* , SSI, em inglês) e testes de tração.

**Métodos** Sete amostras de TP e cinco de ST foram testadas em máquina de tração e simultaneamente avaliadas por SSI. As medidas geradas possibilitaram comparar o comportamento mecânico dos tendões por curva *stress* x *strain* e módulo de cisalhamento (μ) em repouso. Também foi analisada a relação *stress* x μ sob tensão, e testada a relação entre esses parâmetros. Os resultados foram submetidos a análise estatística pelos testes *t* não-pareado com correção de Welch, correlação de Pearson e regressão linear para estimativa do módulo de Young (E).

**Resultados** O μ dos TP e ST em repouso foi, respectivamente, de 58,86 ± 5,226 kPa e 124,3 ± 7,231 kPa, com diferença estatisticamente significativa. O coeficiente de correlação entre *stress* e μ dos TP e ST foi classificado como muito forte. O E calculado dos TP e ST foi, respectivamente, de 19,97 kPa e 124,8 kPa, com diferença estatisticamente significativa.

**Conclusão** O ST foi mais rígido do que o TP nos testes de tração e nas avaliações por SSI. O μ esteve diretamente relacionado com o *stress* a que o tendão é submetido.

**Relevância clínica** Avaliar as propriedades mecânicas dos tendões mais utilizados como enxerto nas cirurgias de reconstrução ligamentar do joelho.

## Introdução

Nas últimas décadas, as propriedades mecânicas dos tendões foram amplamente estudadas, o que proporcionou conhecimento sobre seu comportamento. 1 2 3 4 Recentemente, a avaliação do módulo de elasticidade (E) e da resistência à falha (RF) do tecido tendinoso ganhou importância na literatura. 5 Em 2013, LaCroix et al. 5 evidenciaram íntima relação entre essas propriedades, apesar de serem distintas conceitualmente.

Vários métodos de aferição dessas propriedades foram utilizados nas publicações sobre o tema, mas a maioria contém vieses. 3 6 7 8 9 10 11 12 13 14 Os resultados dos estudos *in vivo* são habitualmente obtidos de maneira indireta, a partir de cálculos que associam imagens de ressonância magnética e ultrassonografia com dados fornecidos por dinamômetro. 10 13 Análises *ex vivo* de tendões de cadáveres humanos e de animais têm sido realizadas por diversos autores. 2 3 6 9 11 12 14 Contudo, a confiabilidade desses estudos é questionável pelo protocolo de preparo dos tecidos cadavéricos estudados. 10 15

Surgiu a necessidade de novos métodos de análise que contemplassem estudos *in vivo* e avaliação direta. Recentemente, a ultrassonografia associada à elastografia vem sendo amplamente aplicada para avaliar a rigidez dos tecidos, pois é capaz de aferi-la de maneira não invasiva, em tempo real, menos dependente da habilidade do operador. 7 16 17 18 19 20

Existem diferentes modalidades de elastografia, e a mais recentemente desenvolvida foi a elastografia por ondas de cisalhamento ( *supersonic shear-wave imaging* , SSI, em inglês). 17 20 21 Nesse método, uma força de radiação acústica gera ondas de cisalhamento no tecido, que são detectadas pelo transdutor ultrassonográfico. 14 17 21 A velocidade dessas ondas e o módulo de cisalhamento (μ) do tecido são fornecidos pelo sistema, e expressa a rigidez. 12 14 17 19 21

O uso da SSI está bem estabelecido para a avaliação de meios isotrópicos, como os tecidos mamário, hepático e tireoidiano. 22 23 24 Apesar de necessitar de validação mais robusta, desde o início da última década a SSI tem sido utilizada no tecido musculoesquelético, um tecido anisotrópico, e o método encontra-se em ascensão promissora. 1 6 7 8 16 18 19 20 25 26 27 Por sua característica anisotrópica, o μ do tendão não apresenta a relação matemática esperada com o E. 14 17 21 Apesar disso, estudos recentes 8 9 11 12 14 25 demonstraram forte correlação entre o μ do tendão obtido pela SSI e seu E calculado pela fase linear do gráfico *stress* x *strain* . Contudo, a maioria desses ensaios utilizou tendões de outras espécies animais, e são de baixa evidência científica. 9 11 12 14

Há na literatura dois estudos 8 25 que comparam o comportamento mecânico de tendões humanos *ex vivo* com a análise elastográfica feita pela SSI. O presente estudo visa avaliar as propriedades mecânicas dos tendões patelar (TP) e semitendinoso (ST), por sua grande relevância e utilização como enxerto nas cirurgias de reconstrução ligamentar do joelho, 28 por meio da SSI e da dinamometria.

O objetivo principal foi obter e comparar o E do TP e ST ao tensionamento, pela avaliação da curva *stress* x *strain* registrada na máquina de tração. Os objetivos secundários foram obter e comparar o μ do TP e ST na avaliação ultrassonográfica em repouso por SSI e analisar a influência da tensão tecidual na avaliação pela SSI, por meio da análise da correlação entre o μ e o *stress* aplicado a esses tendões.

## Materiais e Métodos

## Declaração de ética e desenho experimental

Foi realizado um estudo transversal, aprovado pelo Comitê de Ética em Pesquisa (CEP) da instituição, sob o parecer consubstanciado 1.674.064 (CAAE: 26828914.3.0000.5257), desenvolvido no Laboratório de Biomecânica de 2019 a 2022.

A amostra inicial era composta por 14 amostras de TP e 19 de ST, obtidas de cadáveres humanos congelados enquanto ainda frescos, fornecidos pelo banco de tecidos do Ministério da Saúde (MS). O fornecimento de tais tendões ocorreu após eles serem considerados inaptos para a utilização em cirurgias, principalmente por contaminação em alguma fase do processamento no banco de tecidos. Em sua maioria, o microrganismo detectado foi o *Staphylococcus epidermidis* , bactéria que comumente coloniza a pele de seres humanos.

A distribuição demográfica das amostras está demonstrada nas Tabelas 1 e 2 .

**Tabela 1:** Distribuição demográfica do tendão patelar

Gênero	N	Idade média (anos)
Masculino	5	28,2 (22–35)
Feminino	2	28 (26–30)

**Tabela 2:** Distribuição demográfica do tendão semitendinoso

Gênero	N	Idade média (anos)
Masculino	4	27,5 (22–35)
Feminino	1	22

O uso de tendões humanos congelados enquanto ainda frescos se justificou pelo fato de que tecidos conservados em formol perdem suas propriedades mecânicas. 2 15 Nesse sentido, optou-se por obter o material amostral do MS, pois o ministério tem como protocolo preparar os tecidos musculoesqueléticos sem irradiá-los, o que evita danos às suas características biomecânicas.

Os critérios de inclusão foram: tendões de cadáveres humanos congelados enquanto ainda frescos, com idades entre 20 e 35 anos na data do falecimento, e conservação em *freezer* com temperatura de -80° C no banco de tecidos.

Os critérios de exclusão foram: sinal de doença degenerativa nos tendões, tempo de armazenamento superior a dois anos, presença de rupturas macroscópicas, irradiação dos tendões no preparo pelo banco de tecidos e registro biomecânico ou elastográfico inadequado.

Após a aplicação dos critérios de exclusão e a ocorrência de danos acidentais aos tendões durante o preparo pré-teste, houve perda de 7 amostras de TP e 14 de ST, e restaram 7 amostras de TP e 5 de ST para a análise final.

## Preparo das amostras

As amostras foram mantidas em ambiente refrigerado a -20°C em *freezer* do Laboratório de Imunologia. Para a testagem, os tendões foram descongelados uma hora antes em temperatura ambiente.

O TP foi preparado após o descongelamento da peça anatômica fornecida pelo MS, que consistia no aparelho extensor do joelho por inteiro, com tendão quadricipital, patela, TP e tuberosidade da tíbia. Foram confeccionadas as peças para a testagem com *plugs* ósseos em ambas as inserções do TP, cada uma medindo aproximadamente 1,0 cm em cada dimensão, e a parte tendinosa intermediária, com aproximadamente 1,0 cm de largura ( Fig. 1 ). Cada *plug* ósseo foi perfurado com broca de 2,5 mm, o que gerou orifícios para a passagem de fio Ethibond 5 (Ethicon, Inc., Raritan, NJ, Estados Unidos).

**Fig. 1:** Tendão patelar (TP) preparado para o teste.

O ST teve processamento prévio ao congelamento no banco de tecidos. Seu comprimento foi padronizado desde a junção miotendínea até a sua inserção tibial. Após o descongelamento, foi aplicada sutura tipo Krakow com Ethibond 5 em ambas as extremidades, para otimizar a ancoragem.

Em ambos os tendões, foi utilizado o sistema de fixação à máquina universal de ensaio por garras metálicas ( Fig. 2 ).

**Fig. 2:** Garras metálicas de fixação.

No ST, previamente ao método definitivo de fixação às garras metálicas, foram feitas duas tentativas. Na primeira, os tendões foram presos diretamente às garras, o que provocou dano estrutural macroscópico nas primeiras amostras e soltura de suas extremidades na interface com as garras quando o teste de tração ainda se iniciava, e o método foi abandonado. Em uma segunda tentativa, a ancoragem dos tendões foi realizada amarrando o Ethibond 5 diretamente a tubos plásticos, que foram presos às garras. Mais uma vez, houve perdas por danos estruturais aos tecidos e geração de leitura elastográfica ou biomecânica inadequada, e o método também foi abandonado. Após o fracasso dos testes com esses dois protótipos de ancoragem, infelizmente foram totalizadas 14 perdas de amostras de ST. Finalmente, para gerar fixação mais eficaz entre a garra e o ST, estes foram inseridos em tubos plásticos e fixados a eles com fios Ethibond 5, presos a parafusos convencionais a cavaleiro ( Figs. 3 4 ). Este último método, tido como ideal e definitivo, não causou qualquer dano ou interferência na aquisição dos dados.

**Fig. 3:** Tendão semitendinoso (ST) inserido em tubos e fixado a cavaleiro em parafusos.

**Fig. 4:** Montagem final do ST.

## Elastografia

Para a aquisição das imagens elastográficas, foi utilizado o equipamento Aixplorer (SuperSonic Imagine, Aix-en-Provence, França), com transdutor linear operando na frequência de 6 a 20MHz. Antes de cada teste, o transdutor foi cuidadosamente alinhado no mesmo direcionamento das fibras tendinosas, utilizando o modo B do ultrassom ( Fig. 5A ), garantindo que o μ fosse medido no mesmo sentido da tração longitudinal.

**Fig. 5:** *.* ( **A** ) Tendão alinhado na ultrassonografia (US) modo B. ( **B** ) Região de interesse (RI) no modo elastografia.

O modo elastográfico foi ativado utilizando-se o *preset musculoskeletal* (MSK) adaptado, cuja escala varia de 0 a 800 kPa. A área de mapeamento teve forma retangular, o que permitiu a delimitação do tendão. Para estabilização do mapeamento em cores das imagens elastográficas ( Fig. 5B ), o teste foi iniciado após 10 segundos.

Um braço robótico AUBO i5 (AUBO Robotics, Pequim, China) ( Figs. 6 7 ) foi utilizado na coleta das imagens, e manteve-se o transdutor fixo e imóvel, posicionado sobre a região de interesse (RI). Utilizou-se gel (Ultrex-gel, Farmativa Indústria e Comércio Ltda., Rio de Janeiro, RJ, Brasil) para o acoplamento acústico na superfície do tendão.

**Fig. 6.** Braço robótico mantendo posicionamento do transdutor linear.

**Fig. 7:** Posicionamento do transdutor durante o teste na máquina de tração.

Na análise do μ, foi utilizada uma rotina própria desenvolvida pelo Laboratório de Biomecânica (por meio do programa Matlab R2013a, The MathWorks, Inc., Natick, MA, Estados Unidos). A aquisição das imagens da elastografia foi feita até o momento da saturação do mapeamento. Nesse instante, o vídeo e o tensionamento eram interrompidos.

## Testagem biomecânica

Na realização dos testes de tensionamento, foi utilizada máquina de ensaio de tração Biopdi (São Carlos, SP, Brasil) com célula de carga de 5 kN. A temperatura e a umidade relativa do ar no laboratório durante os testes foram padronizadas e mantidas constantes, a 23 ^°^ C e 50%, respectivamente. O TP e o ST foram presos em suas extremidades às garras de metal que se acoplam à máquina de tensionamento, com uma das extremidades ficando fixa enquanto a outra era progressivamente tracionada a uma velocidade de 1 mm/min, num eixo longitudinal uniaxial.

Os valores da máquina de ensaio foram referentes à posição (mm) e à força (N). Por meio do programa Matlab, os dados foram filtrados no filtro *butterworth* de ordem 6. Foi feito ajuste exponencial de ordem 3, e finalizou-se com *splines* de 0,5. Após o processo, foram calculadas a tensão ( *stress* ) e a deformação ( *strain* ), utilizando o comprimento inicial e a área de secção transversa, medidos anteriormente.

## Análise estatística

Os dados descritivos, como desvio padrão (DP) e média, foram calculados. A normalidade das distribuições foi calculada pelo teste de Shapiro-Wilk. O teste *t* para grupos independentes, com correção de Welch, foi aplicado para comparação do μ em repouso entre TP e ST. O coeficiente de correlação de Pearson foi utilizado para a relação entre o μ sob tensão com o *stress* dos tendões, em instantes da curva *stress* x *strain* . Foi realizada regressão linear sobre as distribuições para a obtenção da medida da inclinação ( *slope* ) da curva. O E foi calculado pela fórmula *slope* ^-1^ , pois optou-se pelo *stress* como variável independente no eixo x. valores de *p* < 0,05 foram considerado significativos. As análises foram realizadas por meio do programa GraphPad Prism (GraphPad Software, Inc., La Jolla, CA, Estados Unidos), versão 7.0.

## Resultados

## Módulo de cisalhamento em repouso

O μ inicial (sem tensionamento) do ST foi superior ao do TP, de forma estatisticamente significativa (ST = 124,3 ± 7,231 kPa; TP = 58,86 ± 5,226; *p* = 0,0059) ( Fig. 8 ).

**Fig. 8:** Módulo de cisalhamento (μ) do TP e do ST, em repouso. **Nota:** * *p* < 0,05.

## Correlação entre módulo de cisalhamento e *stress*

O μ apresentou uma correlação considerada muito forte com o *stress* para ambos os tendões (R = 0,9507; *p* < 0,0001 para o TP; e R = 0,9528; *p* < 0,0001 para o ST) ( Tabela 3 ).

**Tabela 3:** Correlação módulo de cisalhamento (μ) x *stress*

Coeficiente de correlação (R)	N	μ x *stress*	*p*
Tendão patelar	7	0,9507 (0,839–0,985)	< 0,0001
Tendão semitendinoso	5	0,9528 (0,845–0,986)	< 0,0001

## Variação do módulo de cisalhamento no *stress*

A variação do μ do ST e do TP sob *stress* não apresentou diferença estatisticamente significativa (ST: inclinação de 0,664 ± 0,063 kPa; TP: inclinação de 0,872 ± 0,085 kPa; *p* = 0,065). Entretanto, foi observada uma diferença estatisticamente significativa entre o μ dos tendões, especialmente notada em tensões menores (ST: elevação de 116,8–133,3 kPa; TP: elevação de 47,14–69,31 kPa; *p* < 0,0001) ( Fig. 9 ).

**Fig. 9:** Relação *stress* x módulo de cisalhamento (µ). **Nota:** * *p* < 0,05

## Deformação percentual ( *strain* ) sob *stress* e módulo de Young

O ST apresentou maior resistência à deformação do que o TP, com diferença estatisticamente significativa (ST: inclinação de 0,05 ± 0,005; TP: inclinação de 0,008 ± 0,0002; *p* < 0,0001). Uma diferença estatisticamente significativa foi observada com relação ao E calculado dos tendões (ST = 124,8 kPa; TP = 19,97 kPa; *p* < 0,0001) ( Fig. 10 ).

**Fig. 10:** Relação *stress* x *strain* . **Nota:** *p< 0,05

## Discussão

O estudo das propriedades mecânicas dos tendões traz informações sobre sua função, prevenção e tratamento de doenças. 1 3 18 26 27 29 Neste contexto, o ST e o TP se destacam em importância pela sua vasta utilização como enxerto em cirurgias de reconstrução ligamentar. 28

Neste estudo, foram utilizados tendões de cadáveres congelados enquanto ainda frescos, cedidos pelo MS, não danificados por formol ou radiação ionizante, o que possibilitou avaliar a curva *stress* x *strain* e computar a relação entre o *stress* e o *strain* . O ST apresentou maior resistência ao *strain* sob *stress* do que o TP, com diferença estatisticamente significativa ( *p* < 0,0001). Isso também foi observado com o E calculado, que apresentou valor muito superior no ST (ST = 124,8 KPa; TP = 19,97 KPa; *p* < 0,0001). Foi comparado o μ do TP ao do ST, obtidos por meio da avaliação ultrassonográfica em repouso feita por SSI. Novamente, o ST foi mais rígido do que o TP (ST = 124,3 ± 7,231 kPa; TP = 58,86 ± 5,226 KPa; *p* = 0,0059), o que evidencia a concordância entre os resultados obtidos pela SSI e os dos testes de tração. Ainda foi registrada a influência da tensão tecidual na avaliação pela SSI, que evidenciou que, quanto maior o *stress* aplicado, maior o μ registrado em ambos os tendões, havendo correlação muito forte entre os parâmetros (TP: R = 0,9507; *p* < 0,0001: e ST: R = 0,9528;. *p* < 0,0001).

Nas últimas décadas, tem sido demonstrada a relevância da SSI na avaliação dos tendões *in vivo* como uma técnica realizada em tempo real. 7 18 19 20 26 27 29 Uma série de trabalhos 19 29 30 chamaram a atenção para a importância de padronizar a obtenção das imagens, especialmente quanto à posição do membro examinado. Em 2019, foi publicado pela primeira vez estudo 8 que evidencia a relação íntima entre o μ registrado na SSI e o *stress* ao qual é submetido um tendão humano não formolizado. No entanto, tal trabalho utilizou apenas um espécime, e apresentou baixo nível de evidência. No presente estudo, repetiu-se essa testagem com 2 tipos de tendões (TP e ST), e foram utilizadas 12 amostras; evidenciou-se correlação muito forte entre essas duas variáveis, com significância estatística. Isso traz uma nova luz sobre o uso da avaliação de tendões por SSI. A partir deste momento, torna-se de fundamental importância controlar a ação muscular atuante sobre o tendão estudado, pois esta pode afetar significativamente o registro elastográfico.

A literatura atual demonstra consistentemente a relação entre o E e o μ, embora não respeitando a estimativa matemática clássica. 9 12 Recentemente, em 2023, Brandão et al. 25 estudaram 5 TPs e 11 tendões aquileus de cadáveres humanos congelados enquanto ainda frescos, e encontraram forte correlação entre a variação do μ registrado na SSI com o E calculado pela curva *stress* x *strain* no teste biomecânico. No presente trabalho, foi observada maior rigidez do ST do que do TP, tanto na SSI quanto no teste de tração, com significância estatística. Isto sugere que a SSI é capaz de aferir as propriedades mecânicas dos tendões de forma confiável, comparável ao padrão-ouro.

No contexto da utilização do TP e do ST como enxertos para reconstrução ligamentar, é interessante que esses tendões não só apresentem uma grande RF, como também características elásticas semelhantes às do ligamento nativo. Estudos prévios 5 11 revelaram correlação estatisticamente significativa entre o μ e a RF em tendões animais normais. Da mesma forma, foi descrita redução do μ e da RF em tendões doentes ou danificados quimicamente. 11 14 Portanto, a SSI pode ser particularmente útil na avaliação de tendões e na tomada de decisão na escolha do enxerto. No entanto, nenhum destes trabalhos realizou tais testagens em tendões humanos.

Os achados deste estudo são semelhantes aos de Fontenelle et al. 19 em 2018, em que o μ do ST foi superior ao do TP em estados relaxado e tensionado, *in vivo* . Isto pode sugerir a escolha do ST na reconstrução de uma estrutura mais rígida.

É importante ressaltar algumas limitações do presente ensaio. Apesar de terem sido obtidos valores consistentes para o E pelo cálculo da inclinação da curva *stress* x *strain* , 19 os tendões não foram levados até a falha. Os testes foram interrompidos ao atingir a saturação da SSI, sendo possível que ainda não tivesse sido atingida a região de deformação elástica linear do tendão, o que comprometeria a estimativa do E. Além disso, apesar de terem sido tomados todos os cuidados possíveis, a fixação dos tendões às garras metálicas da máquina de tração se revelou particularmente difícil quando não havia um *plug* ósseo. Isso pode gerar micromovimentos na interface tendão-garra, e uma subestimação do registro de deformação obtido. Futuros estudos devem considerar isso.

Por último, o enxerto mais adequado depende do comportamento mecânico não só do tendão, como também dos ligamentos a serem substituídos. As próximas pesquisas nesta área devem dedicar particular atenção à análise biomecânica desses ligamentos.

## Conclusão

O ST foi mais rígido do que o TP, tanto no teste de tração quanto na avaliação pela SSI em repouso e sob tensão, sendo que o μ revelou relação direta com o *stress* ao qual o tendão é submetido durante sua avaliação.
